# Dual Imaging Approach Using Laser-Induced Fluorescence and Hyperspectral Reflectance for Automated Sorting of White Polyamide in Mixed Waste

**DOI:** 10.1007/s10895-025-04575-6

**Published:** 2025-11-08

**Authors:** Mohamed Ebrahem, Alaaeldin Mahmoud, Yasser H. El-Sharkawy

**Affiliations:** Optoelectronics and Automatic Control Systems Department, Military Technical College, Kobry El-Kobba, Cairo, Egypt

**Keywords:** White polyamide, Automated sorting, Diffuse reflectance spectroscopy, laser-induced fluorescence, Hyperspectral imaging, Recycling technology

## Abstract

In automated recycling systems, accurately sorting mixed-material waste remains a significant challenge, particularly when materials such as wood, metals, and polymers exhibit similar visual appearances. White polyamide polymer is widely used in industrial components due to its durability and chemical resistance, yet its recovery from waste streams is often hindered by optical similarities to light-colored wood and oxidized or coated metals. This study presents a dual imaging approach that combines laser-induced fluorescence (LIF) using ultraviolet excitation with hyperspectral imaging (HSI) of diffuse reflectance under broadband illumination (400–1000 nm). The fluorescence experiments revealed that the white polyamide polymer exhibited a strong significant wavelength at approximately 740 nm, distinctly separating it from wood and metal, alongside a less prominent secondary response at 443 nm. However, in the visible range from about 480 to 630 nm, the fluorescence responses of wood and polymer substantially overlapped, underscoring the importance of targeting the near-infrared (NIR) emission for effective polymer discrimination. Complementary diffuse reflectance HSI data analyzed through histogram techniques identified optimal wavelengths near 480 nm and 840 nm that enhanced contrast between the polymer, wood, and metal, guiding the design of simplified multispectral systems. This dual-modality imaging strategy integrates molecular fluorescence sensitivity with detailed reflectance profiling to achieve improved material discrimination, paving the way for practical, automated sorting solutions. The insights gained also support the future development of conventional aerial camera systems equipped with optimized filters and illumination sources to monitor and manage polymer waste accumulation on larger environmental scales.

## Introduction

The rapid acceleration of global industrialization and consumer markets has led to an unprecedented rise in the generation of solid waste, posing significant challenges to environmental sustainability and public health [1, 3]. Among the diverse categories of waste, synthetic polymers represent one of the most problematic classes due to their remarkable durability, chemical inertness, and extensive applications across sectors ranging from packaging and construction to automotive and electronics [4, 6]. White polyamide polymers are widely utilized in mechanical systems, plumbing infrastructure, and precision-engineered components because of their high tensile strength, thermal stability, and resistance to corrosive chemicals [7, 8]. However, these same properties that make polyamide invaluable in industrial contexts render it equally persistent in post-consumer and industrial waste streams [9, 10]. Efficiently recovering and recycling such polymers is therefore critical for reducing landfill accumulation, conserving natural resources, and supporting the global shift toward circular economy principles [11, 12].

Despite the pressing need for high-throughput recycling systems capable of isolating valuable polymers like white polyamide from complex waste mixtures, current sorting technologies face notable shortcomings [13, 14]. Traditional mechanical sorting techniques, such as manual picking or density-based flotation, are labor-intensive and error-prone, often resulting in cross-contamination that diminishes the quality of recycled outputs [15, 16]. More sophisticated approaches based on NIR spectroscopy have become standard in many material recovery facilities because they exploit characteristic vibrational overtones of C–H, N–H, and O–H bonds to differentiate among common polymer types [17, 19]. However, these methods often fail when dealing with visually similar or coated materials [20, 21]. White or lightly colored polymers, in particular, tend to exhibit weak or ambiguous NIR absorption bands that overlap with signals from other organic substrates such as wood, or from oxidized metal surfaces whose corrosion layers scatter and absorb light in complex ways [22, 23]. Consequently, this can obscure or distort the intrinsic spectral features necessary for reliable identification, exacerbating sorting errors and reducing the economic viability of polymer recovery streams [24]. This challenge is especially pronounced in the case of white polyamide, whose diffuse optical reflectance characteristics can closely mimic those of polished or lightly weathered wood as well as oxidized or coated iron surfaces [25, 26]. As a result, conventional NIR-based or broadband color imaging systems frequently misclassify these materials, leading to substantial contamination in recycled polymer streams, reducing the overall value of recovered products, and ultimately undermining broader sustainability targets [27, 28]. To address these persistent obstacles, this study introduces a dual imaging strategy that integrates LIF under ultraviolet excitation with HSI of diffuse reflectance spanning the visible to NIR spectral range. This integrated approach is motivated by the fundamentally different optical principles underlying each modality, which together provide a more comprehensive means of material discrimination than either could achieve alone. Laser-induced fluorescence operates by illuminating a sample with a focused laser beam of a specific excitation wavelength, typically in the ultraviolet or visible range, to promote electrons in the molecular structure to higher energy states [29, 30]. As these electrons relax back to ground states, the material emits light at longer wavelengths that are characteristic of its molecular composition [31]. Polymers such as white polyamide, rich in amide linkages and sometimes aromatic stabilizers, often exhibit distinct fluorescence emissions that differ from the signals produced by lignin-containing wood or by inorganic metal surfaces [32, 34]. In contrast, hyperspectral imaging captures detailed spatially resolved reflectance spectra across hundreds of contiguous wavelengths for each pixel in an image [35, 36]. This technique provides a rich dataset that encodes variations in surface composition, scattering properties, and microstructural features [37]. Under broadband white light illumination, hyperspectral systems can reveal subtle differences in how wood fibers scatter light compared to the smoother surfaces of polymers or the crystalline or corroded textures of metals [38]. However, hyperspectral reflectance alone is not universally reliable. When materials have overlapping or relatively featureless spectral signatures, or when coatings and surface fouling homogenize reflectance characteristics, even advanced multivariate analysis may struggle to achieve robust classification [39, 40]. These limitations underscore the importance of integrating complementary modalities. In particular, identifying the most informative spectral bands from both fluorescence emission and diffuse reflectance spectra provides a foundation for developing simplified multispectral or filter-based imaging systems that retain the discriminative power of full-spectrum approaches. Instead of requiring expensive full-range hyperspectral cameras or high-power tunable lasers scanning across multiple excitation wavelengths, targeted systems can be developed that focus on specific spectral bands demonstrated to offer maximum discriminatory power. This strategy not only reduces hardware complexity and cost but also enables faster image processing and real-time decision-making, supporting the stringent throughput requirements of industrial conveyor-based sorting lines. Ultimately, this integrated optical framework represents a significant advancement toward intelligent, automated sorting solutions capable of effectively handling challenging waste streams containing visually and spectrally similar materials such as white polyamide, wood, and metal debris. By bridging the gap between molecular fluorescence analysis and broadband structural reflectance characterization within a unified system, this approach also paves the way for the deployment of aerial or drone-based monitoring platforms equipped with optimized multispectral sensors. Such systems could enable large-scale environmental surveillance of polymer waste accumulation in landfills, waterways, and industrial sites, thereby improving environmental management and enforcement of recycling standards. Through these combined methodologies, the work presented in this study directly contributes to more sustainable material lifecycles, reduced ecological footprints, and progress toward achieving global circular economy objectives.

## Materials and Methods

**Fig. 1:**
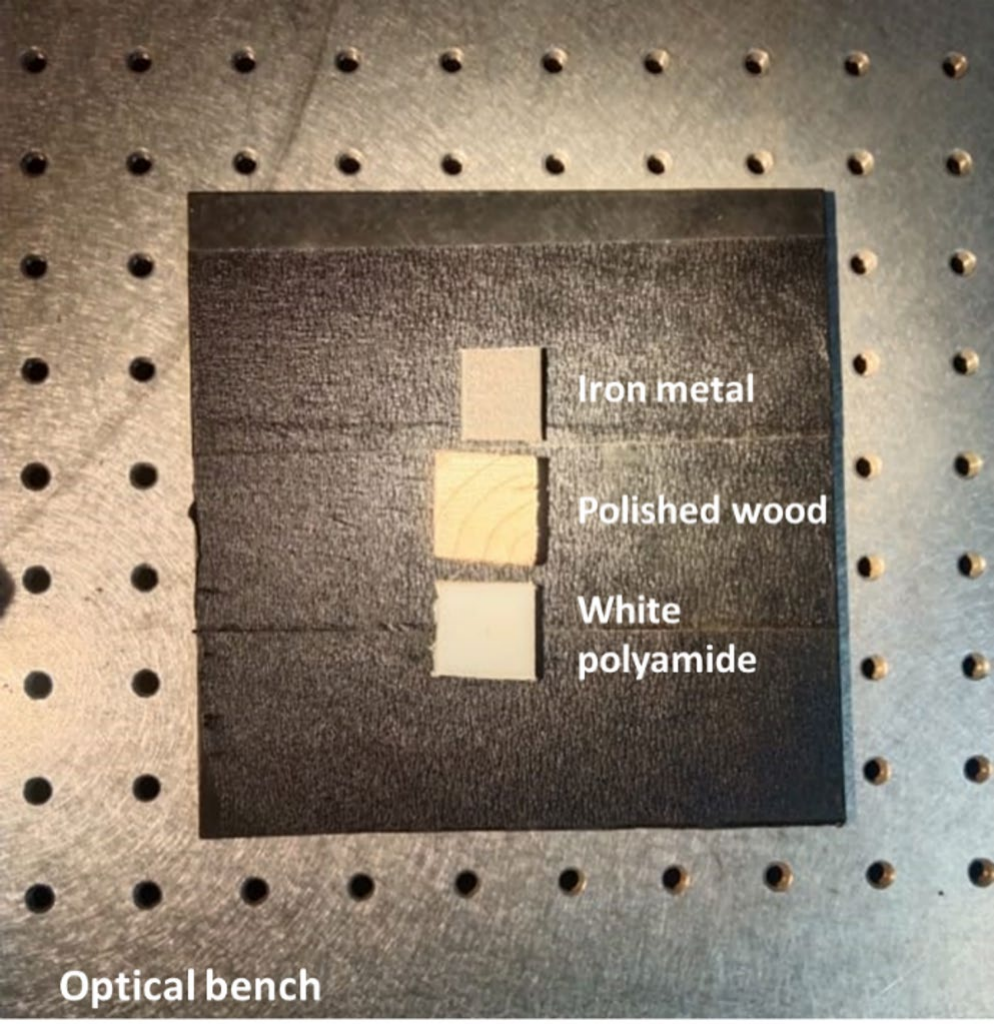
Visual comparison of the three materials investigated in this study: white polyamide polymer, polished natural wood, and iron metal

Three representative materials were selected to simulate realistic and challenging sorting conditions typically encountered in automated recycling environments: white polyamide, polished softwood, and iron metal. These materials were deliberately chosen due to their striking visual similarity under standard illumination, which often results in substantial ambiguity when using conventional color-based sorting systems. Figure 1 illustrates this challenge by showing the comparable appearance between the white polyamide polymer and light wood, as well as the smooth reflective surface of iron. To ensure reproducibility and minimize thickness-dependent artifacts in fluorescence measurements, the polymer and wood were prepared as flat specimens with identical dimensions of 20 mm × 20 mm × 3.0 mm (± 0.2 mm) and matte-finished surfaces for uniform optical conditions. Due to the inherent size limitations of the available iron sample, its dimensions were slightly smaller (approximately 20 mm × 18 mm × 3.0 mm), while maintaining the same thickness to ensure consistent optical response during measurement. The polyamide sample consisted of unfilled PA6 (resin grade BASF Ultramid^®^ B27) with no additional additives or surface coatings. The wood sample was softwood, kiln-dried, untreated, and mechanically polished to a smooth matte finish, while the metal was iron (low-carbon steel), mechanically polished to reduce surface glare and free of coatings or rust. This close resemblance highlights a persistent problem in modern automated recycling systems that largely rely on visual cues and RGB cameras, making it difficult to reliably distinguish between similar-looking waste components. Polyamide was specifically chosen in its white form for this investigation because of its widespread use in engineering applications. Valued for its mechanical strength, affordability, and resistance to chemical and environmental stresses, white polyamide is extensively employed in producing industrial parts, piping systems, and consumer goods. Accurate identification of such polymers is crucial in recycling streams to ensure material purity, avoid contamination, and enable effective circular economy practices. Metal (iron) and wood, meanwhile, frequently coexist with polymers in municipal and industrial waste, demanding precise sorting strategies to maximize recovery rates. By focusing on these three visually similar but compositionally distinct materials, this study aimed to test advanced sensing approaches under conditions that closely mimic real-world industrial constraints.

**Fig. 2:**
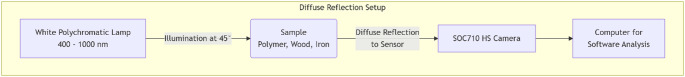
Schematic of the diffuse reflectance HSI setup. A white polychromatic lamp (400–1000 nm) illuminates the samples, and reflected light is recorded by the SOC710 HS camera

**Fig. 3:**
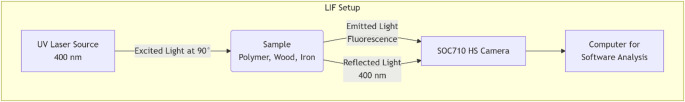
Schematic of the LIF imaging setup. A UV laser source excites the samples (polyamide, wood, and iron), inducing fluorescence emission

To tackle this challenge, two complementary optical sensing modalities were employed: LIF approach under ultraviolet excitation (400 nm, beam diameter at 40 cm ≈ 0.8 mm) and diffuse reflectance spectroscopy using hyperspectral imagery under broadband illumination (Derungs, 400–1000 nm 20 P SX). Both methods were implemented to record detailed spectral information across the visible to NIR range (400–1000 nm), enabling a robust exploration of intrinsic optical contrasts beyond simple visual appearance. For the diffuse reflectance spectroscopy experiment (illustrated in Fig. 2), a broadband white polychromatic lamp emitting across 400–1000 nm was used to uniformly illuminate the samples. Reflected light from the surfaces of the polyamide, wood, and iron was then captured using a SOC710 HS hyperspectral camera, which is a push-broom scanner designed for high-resolution spectral imaging in the VNIR range. The SOC710 camera features a spectral resolution of approximately 5 nm with 128 bands across its range, enabling precise detection of subtle reflectance differences. This line-scanning imager captures 520 pixels per line, achieving a spatial resolution finer than 40 μm and a spectral resolution of 696 lines per data cube. Data were transferred to a connected computer system equipped with spectral processing software for subsequent analysis. This setup allowed the acquisition of reflectance spectra from each material, generating detailed hyperspectral data cubes where each pixel contains a complete spectrum. The diffuse reflection modality is critical because it reveals wavelength-dependent scattering and absorption properties tied to the material’s microstructure and intrinsic molecular bonds. Prior to initiating the experimental measurements, a rigorous calibration procedure was performed to optimize the signal-to-reflectance ratio and ensure data reliability. HS imagery was first corrected using a standard two-point calibration method based on white and dark reference images. A Spectralon^®^ white reference panel (≈ 99% reflectivity) was used to capture the system maximum response under identical illumination conditions, serving as the bright reference. For the dark reference, a non-reflective opaque cover was placed completely over the camera lens to record the sensor’s baseline response in the absence of light. These two reference images were then used to correct the raw HS data according to the following equation [41]:

1 $$\:{R}_{fc}=\frac{{R}_{oc}-{R}_{Dc}}{{R}_{Bc}-{R}_{Dc}}$$

where *R*_*fc*_ represents the calibrated reflectance image, *R*_*oc*_ is the original raw image, *R*_*Dc*_​ is the dark reference image, and *R*_*Bc*_​ is the bright (white reference) image. This normalization process effectively removes system bias and illumination non-uniformity, yielding accurate relative reflectance values for all subsequent measurements. In the LIF configuration (shown in Fig. 3), the same set of materials was excited using a UV laser source, selected for its ability to stimulate characteristic electronic transitions within organic molecules. When illuminated by the UV laser (50 mWatt), materials such as polyamide and wood can exhibit fluorescence emissions at longer wavelengths. During fluorescence measurements, no additional clean-up or blocking filters were used; instead, the camera position was angled to minimize laser scatter. This emitted light was captured by the SOC710 HS hyperspectral camera positioned at an angle of 90ᵒ to collect fluorescence while minimizing the collection of direct laser scatter. The hyperspectral data was similarly processed on a computer workstation. The LIF technique is particularly valuable for capturing material-specific luminescence signatures, arising from unique chromophoric groups within the polymer or organic lignin structures in wood. In contrast, metals generally lack strong fluorescence under UV excitation, providing a means of discrimination.

Each experiment, both diffuse reflectance and LIF, was repeated ten times to ensure consistency and enhance the reliability of the observed outcomes. Samples remained stationary during acquisitions, and the camera was not moved, ensuring spatial consistency. This iterative measurement process accounted for minor variations in sample positioning, and illumination uniformity, leading to statistically robust data sets. The exposure time was automatically optimized by the SOC710 operating software to prevent saturation. Data acquisition and calibration (including white and dark reference corrections) were performed using the SOC710 operating software with HSAnalysis™ Data Analysis and Calibration Software (HS-Analysis 2XL, Surface Optics Corp.). Subsequent preprocessing, visualization, and advanced signal processing were carried out using MATLAB 2024a Software. No smoothing or interpolation was applied to the raw spectral data except for normalization and conversion to dB for clarity.

## Experimental Results and Analysis

### Analysis of Laser-Induced Fluorescence Response

**Fig. 4:**
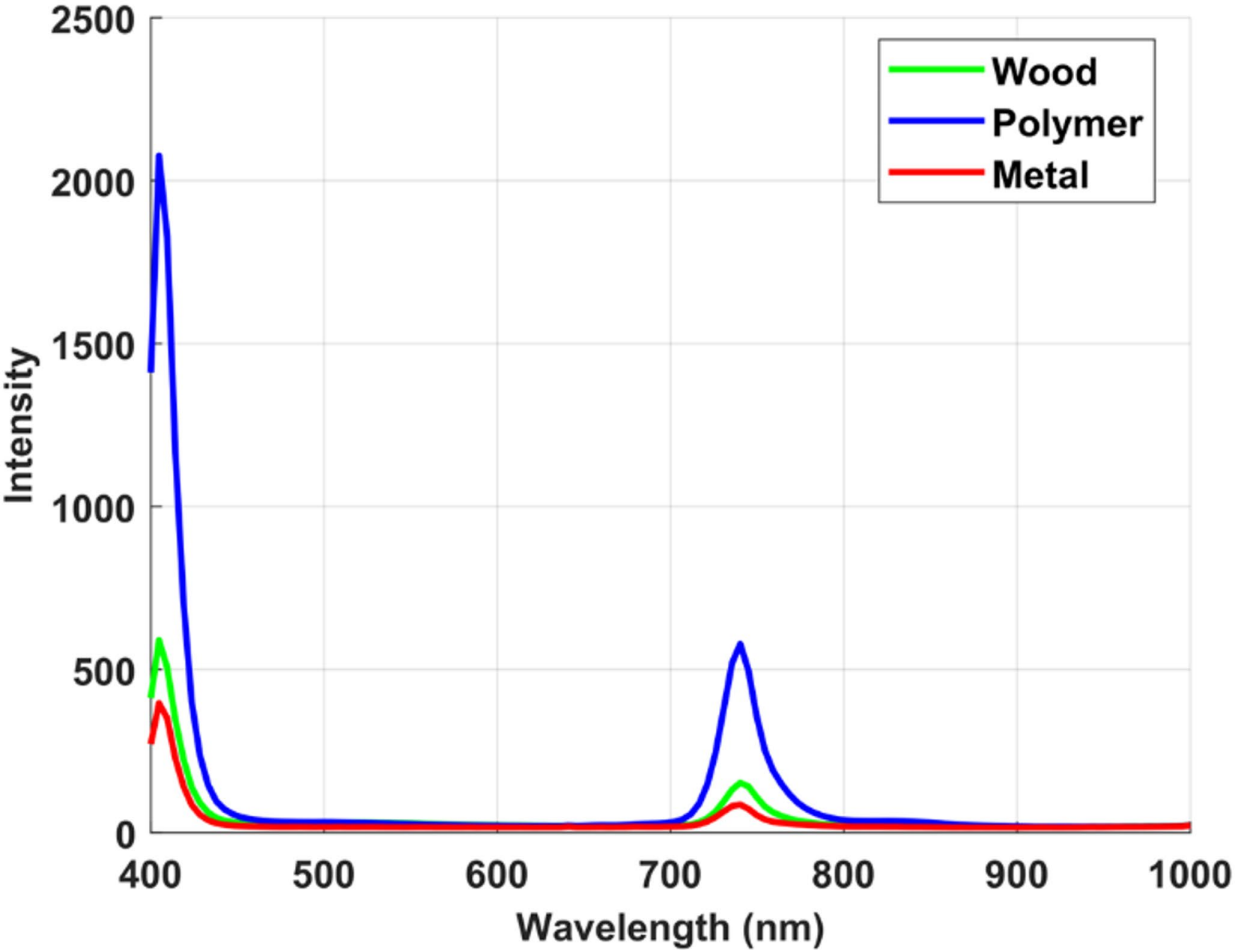
Average raw laser-induced fluorescence (LIF) intensity curves for white polyamide polymer, wood, and metal under UV excitation

**Fig. 5:**
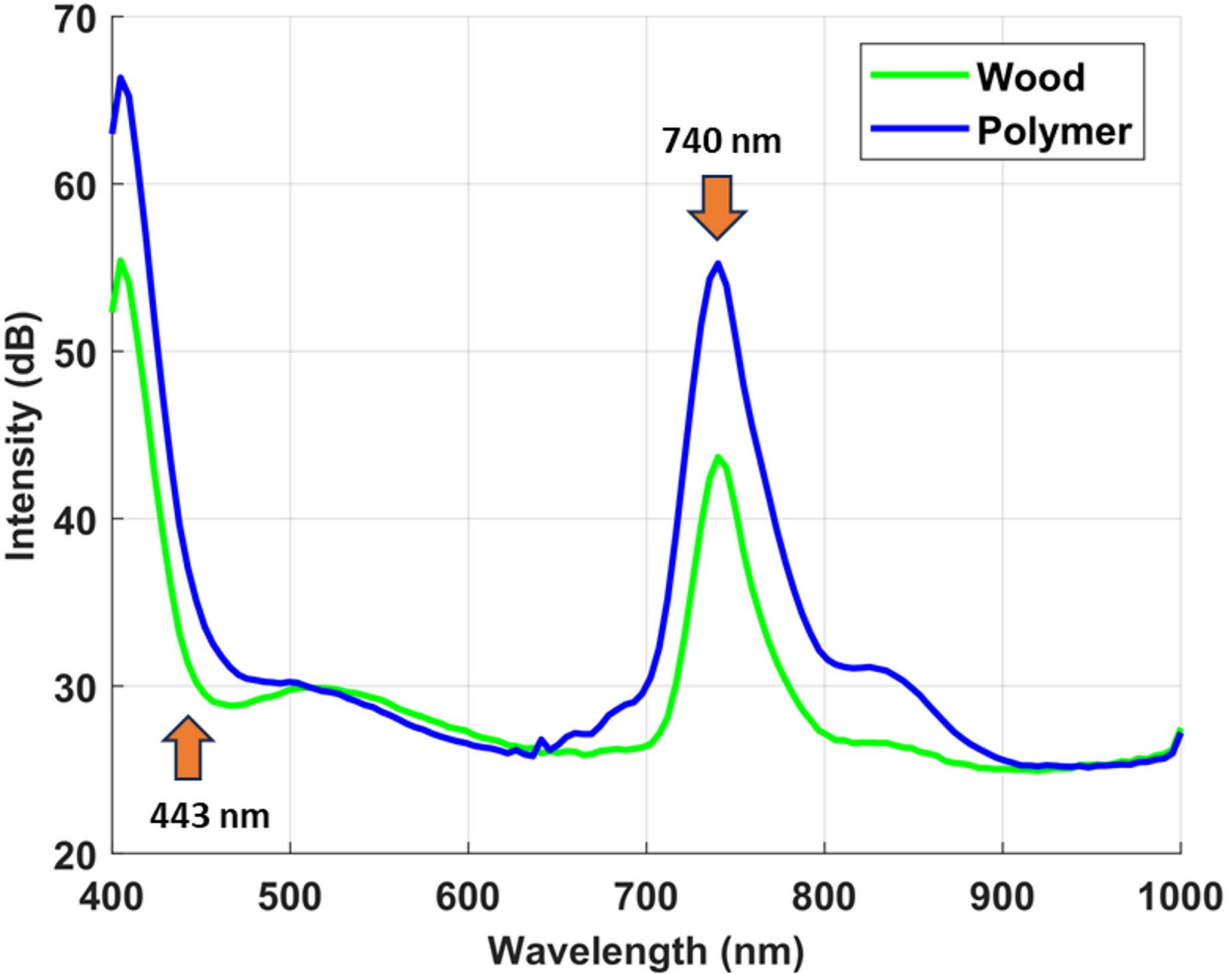
Average normalized LIF intensity curves expressed in decibels (dB) for white polyamide polymer, and wood, highlighting relative emission strengths and key spectral differences used for material discrimination

To rigorously evaluate the ability of the proposed dual imaging approach to differentiate visually similar materials, we first examined the spectral fluorescence responses of the selected samples under ultraviolet laser excitation. The LIF modality provides a powerful means of probing intrinsic molecular signatures that are often invisible to conventional imaging, thereby offering a unique avenue for material discrimination. To enable consistent cross-material comparisons and enhance interpretability of fluorescence differences, all measured intensity values were first normalized to the maximum recorded intensity within the dataset. This step eliminates absolute magnitude variations and ensures that comparisons focus on relative spectral patterns rather than raw intensity differences. The normalized values were then expressed on a logarithmic decibel (dB) scale. This transformation is applied solely for visualization and comparative purposes and does not alter the inherent relative differences between materials. Furthermore, because all samples were solid pieces of comparable thickness and measured under identical optical conditions, concentration-dependent effects were inherently controlled, ensuring that normalization and scaling do not introduce artifacts into cross-material comparisons. By plotting the spectral signatures in dB, even small deviations between wood and polymer across wavelengths become more noticed, enabling clearer identification of regions with maximum contrast for material discrimination. Figures 4 and 5 display the mean raw intensity and the corresponding normalized LIF intensity curves, expressed in decibels (dB), for the white polyamide polymer and the natural wood sample over the 400 to 1000 nm spectral range. These measurements capture the characteristic emission profiles arising from specific electronic transitions within the molecular structures of each material.

**Fig. 6:**
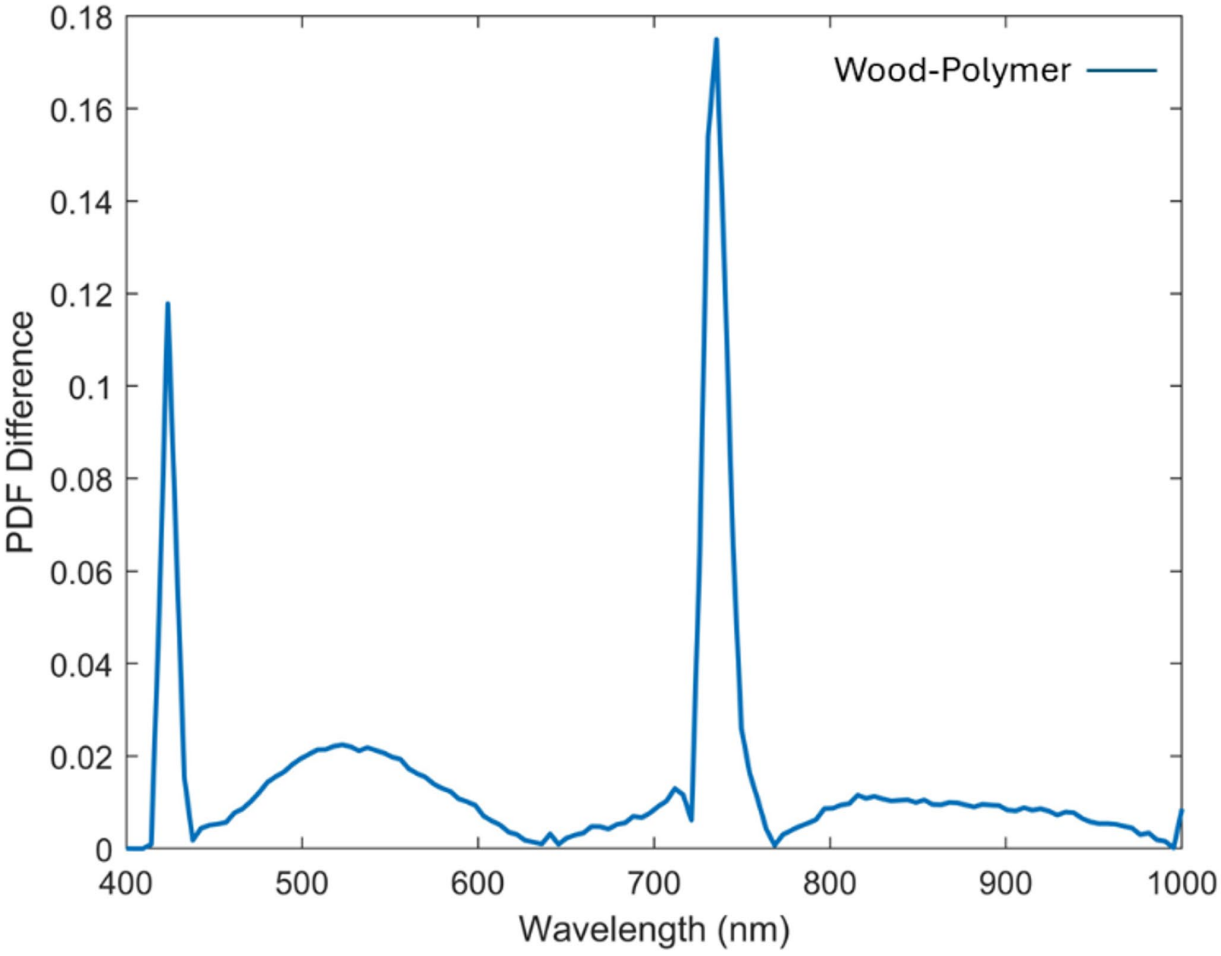
Probability density function difference of fluorescence intensity between the examined polyamide polymer and wood

As depicted in Fig. 4, although an emission feature near 740 nm was observed across all studied samples, its diagnostic value lies in the intensity contrast rather than in spectral shape. The white polyamide polymer exhibited a consistently strong emission at this wavelength, while wood showed only a weak residual signal, likely due to long-wavelength lignin fluorescence tails, and metal remained essentially at baseline, with any apparent signal most plausibly arising from surface scattering rather than true emission. The apparent similarity in spectral shape across materials can be attributed to instrument response, whereas the material-specific differences are preserved in emission intensity. By normalizing all spectra to the dataset maximum and expressing them on a logarithmic dB scale, subtle deviations became more noticeable, enabling clearer separation between polymer and wood. The proposed analysis therefore relied on the dB-transformed spectra to emphasize the regions of maximum contrast. As shown in Fig. 5, the white polyamide polymer exhibits two prominent fluorescence emission peaks. The most significant occurs near 740 nm, representing a strong NIR fluorescence response that is relatively absent in both wood and metal, providing a clear and highly selective spectral marker for this polymer. This pronounced emission is attributed to the inherent molecular structure of polyamide, which includes chromophoric groups capable of absorbing the UV excitation energy and re-emitting in the NIR band. At this wavelength, the fluorescence intensity for polyamide reaches approximately 55 dB, compared to 43 dB for wood, establishing a substantial 12 dB margin that significantly enhances the reliability of discrimination. Such a strong peak at 740 nm forms a robust basis for distinguishing the polymer from both organic and metallic waste streams in automated sorting processes. A secondary significant wavelength is observed at approximately 443 nm, again more intense for the polymer than for the wood sample. This feature, while less dominant than the 740 nm band, nonetheless contributes additional contrast in the visible spectrum, which can be particularly valuable when designing filter-based detection schemes. However, Fig. 5 also reveals a region of spectral overlap between roughly 480 nm and 630 nm, where the emission intensities of the polymer and wood converge closely. While these wavelengths provide limited discriminative power, they are not required for effective classification. Instead, the strong NIR emission at 740 nm represents a more robust feature for automated sorting. Focusing on such distinctive wavelengths ensures optimal discrimination without relying on ambiguous regions. Notably, no appreciable fluorescence was detected for the iron metal under the same UV excitation conditions, confirming that metals do not exhibit similar emission mechanisms and thus remain effectively dark in the fluorescence domain. This absence further simplifies the material separation task, as any strong emission signature can be confidently attributed to non-metallic components. The spectral intensity distributions of each material were modeled using a normal probability density function (PDF), parameterized by the calculated mean and standard deviation. These parameters, summarized in Table 1. To confirm the robustness of 740 nm as a discriminative feature, a PDF analysis was applied (Fig. 6), demonstrating minimal overlap between polyamide polymer and wood at this band compared to the weaker shoulder near 830 nm, thereby validating 740 nm as the optimal wavelength for material separation.

**Table 1:** Mean and standard deviation of normalized fluorescence intensity values for wood, white polyamide polymer, and metal under LIF modality

Material	Mean	Standard deviation
Wood	0.050508	0.1453
polyamide polymer	0.046954	0.1466
metal	0.041441	0.14311

**Fig. 7:**
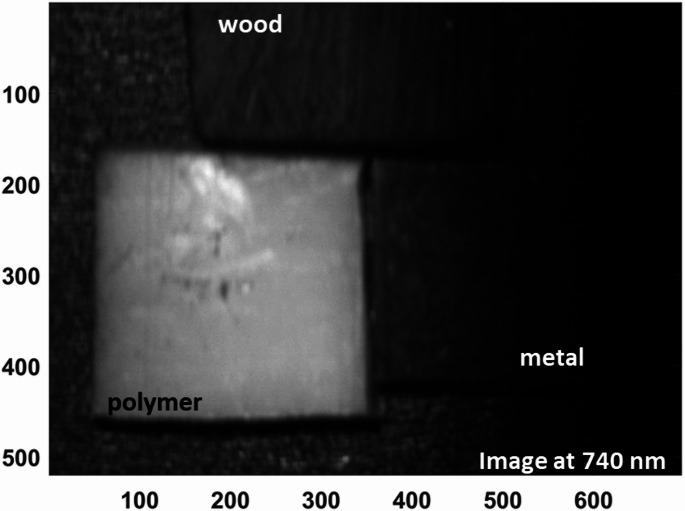
Hyperspectral fluorescence image at 740 nm showing strong emission from the white polyamide polymer compared to wood and metal

**Fig. 8:**
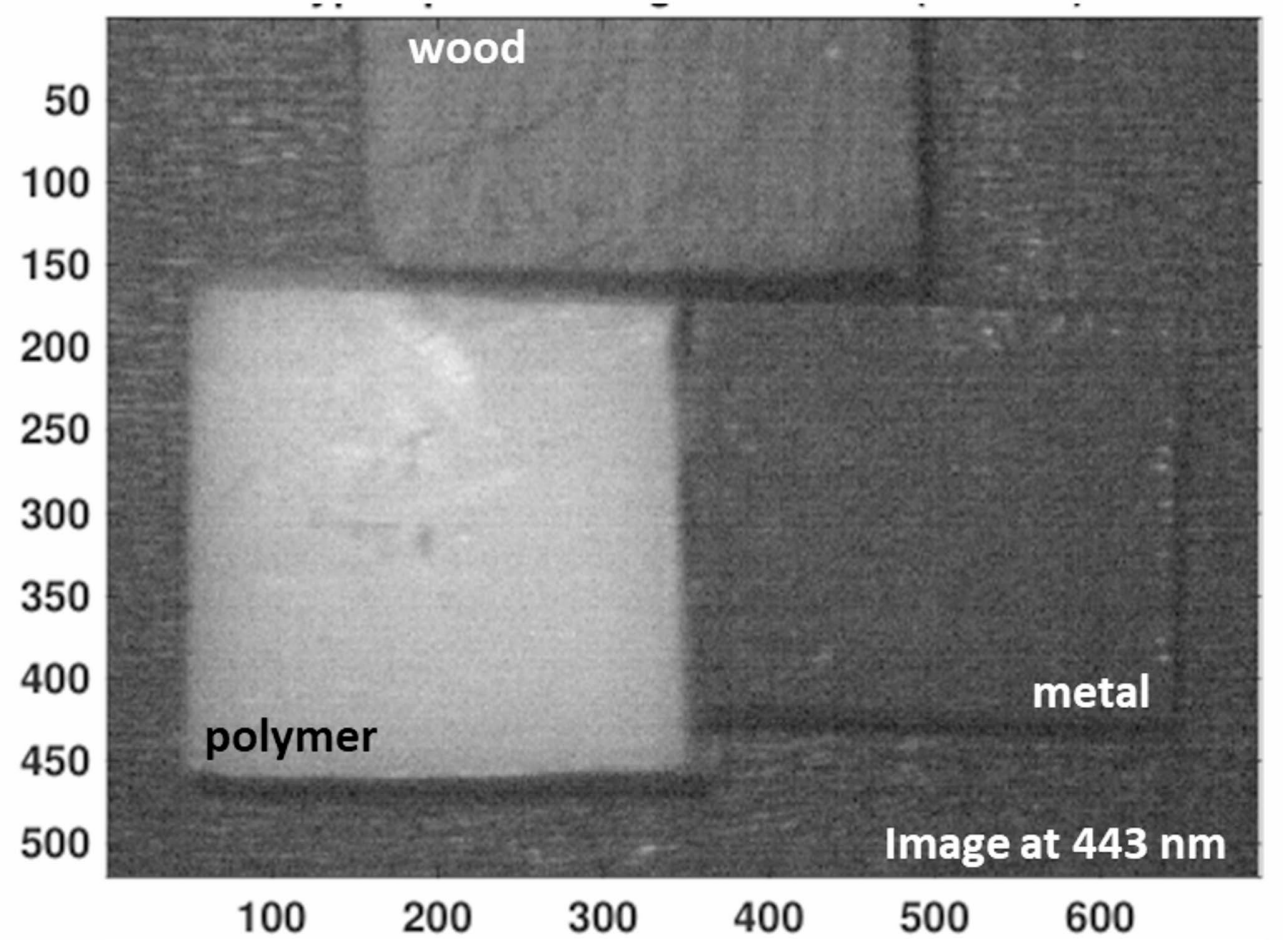
Hyperspectral fluorescence image at 443 nm highlighting a secondary significant wavelength from the polymer, which also provides contrast relative to wood and metal

**Fig. 9:**
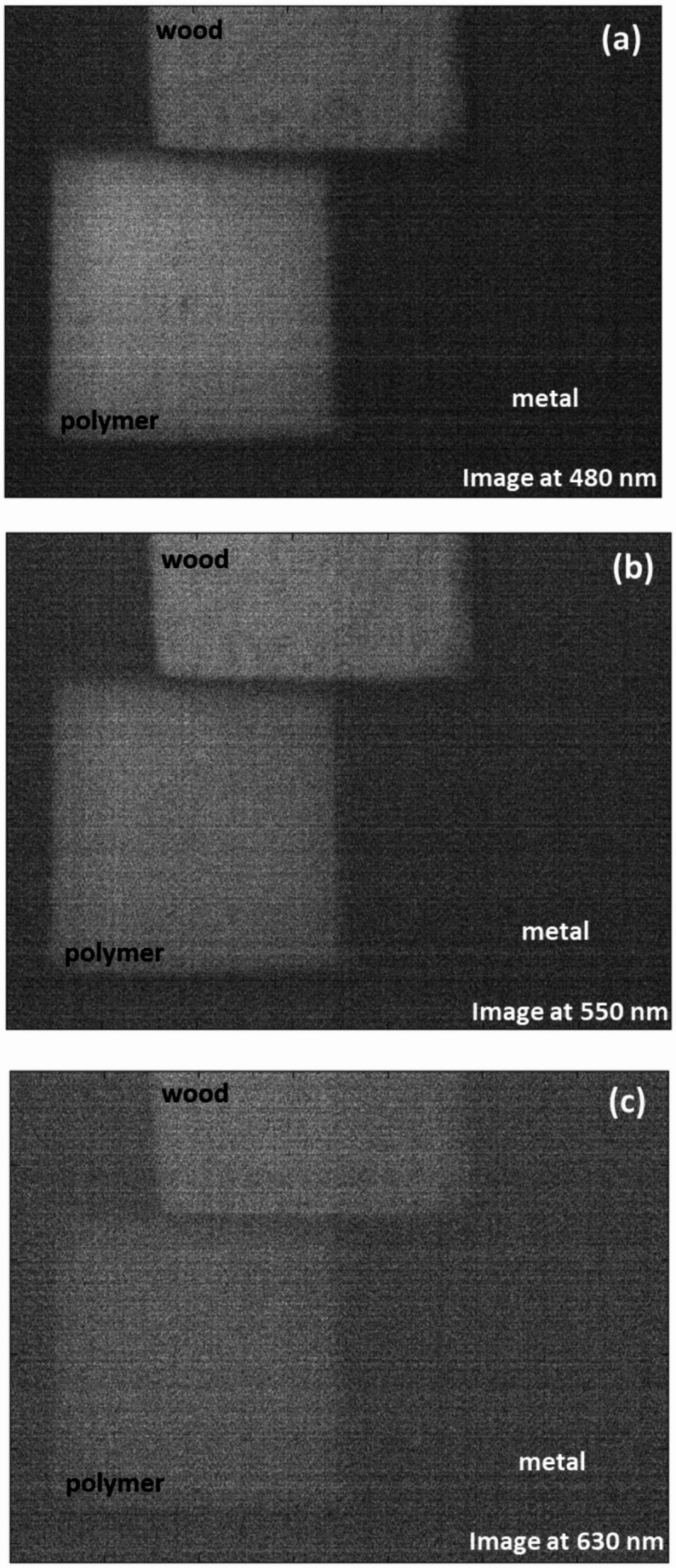
Fluorescence images illustrating significant spectral overlap between wood and polymer, with minimal intensity differences at (**a**) 480 nm, **b** 550 nm, and (**c**) 630 nm

As shown in Fig. 6, The probability density analysis confirms that the primary NIR peak at 740 nm offers the highest separability between white polyamide and wood, making it the most reliable feature for discrimination. However, the secondary significant wavelength observed in the visible region, around 443 nm, also shows a measurable difference between polyamide and wood, as reflected in its distinct distribution in the probability density plot. While this peak is less dominant compared to 740 nm, it provides supplementary discriminative power and could be valuable for designing filter-based detection systems that operate in the visible spectrum (400–700 nm), particularly when NIR sensing is not available or needs to be complemented choice. The effectiveness of LIF imaging in distinguishing metal, wood, and polymer was systematically evaluated by analyzing HS images acquired at key wavelengths of interest. As illustrated in Fig. 7, the fluorescence emission at 740 nm is strongly pronounced for the white polyamide polymer, enabling clear differentiation from both wood and metal, which exhibit substantially lower emission intensities at this wavelength. Additionally, a secondary fluorescence response from the polymer is observable at 443 nm, as depicted in Fig. 8, further supporting its distinct spectral behavior in the blue region. However, the analysis also revealed that in the visible range, particularly between 480 nm and 630 nm, the fluorescence signatures of wood and polymer become increasingly similar. This convergence is evident from the reduced spatial contrast in the hyperspectral images sampled at 480 nm, 550 nm, and 630 nm, presented in Fig. 9a, b, and c, respectively. These images highlight substantial spectral overlap, underscoring the challenge of reliably separating wood and polymer based solely on fluorescence within this region. This finding reinforces the necessity of incorporating multiple spectral bands, including longer wavelengths where the polymer demonstrates dominant emission, to achieve robust material discrimination in automated sorting systems.

### Identification Analysis of Diffuse Reflectance Spectra

**Fig. 10:**
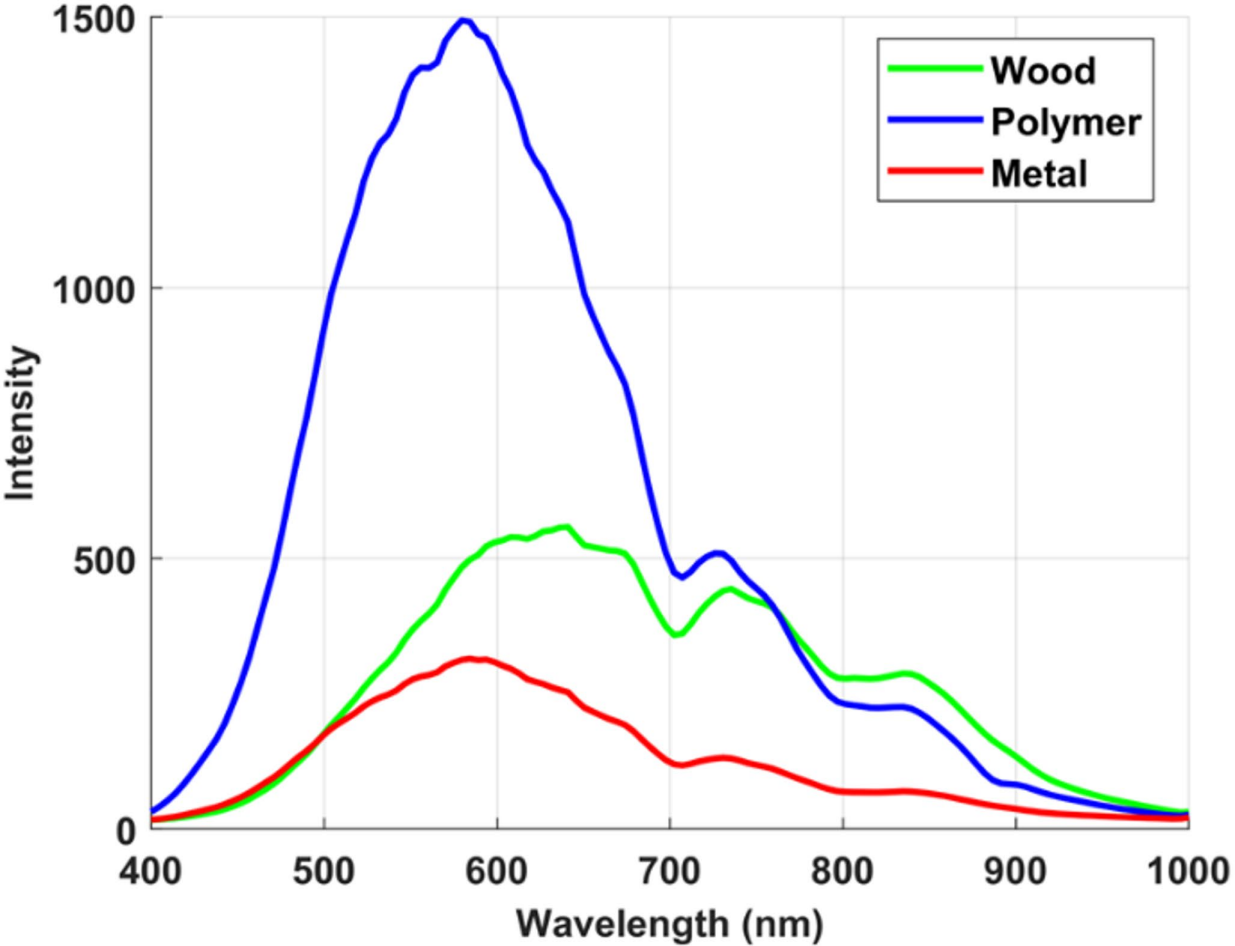
Average raw diffuse reflectance intensity curves for white polyamide polymer, wood, and metal captured by the HIS system, illustrating characteristic spectral profiles across the 400–1000 nm range

**Fig. 11:**
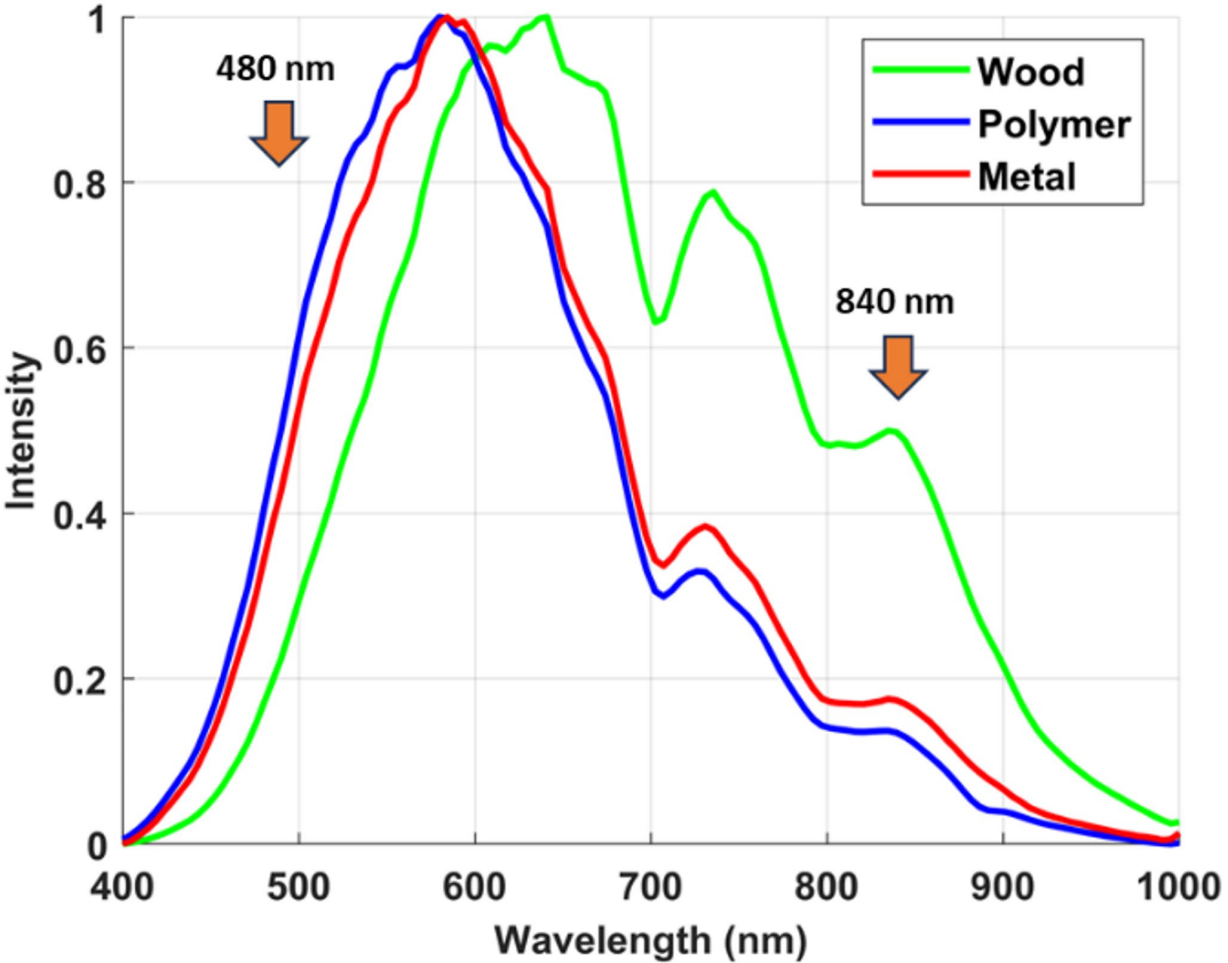
Average normalized diffuse reflectance spectra for white polyamide polymer, wood, and metal, emphasizing relative reflectance differences critical for identifying optimal wavelengths in automated material sorting

Following the LIF investigation, diffuse reflectance spectroscopy was employed using a HSI system to characterize how the selected materials reflect broadband white light across the visible and NIR spectrum. This modality leverages intrinsic differences in optical absorption and scattering caused by the microstructure and chemical composition of each material. The SOC710 HS camera was utilized to capture high-resolution reflectance data spanning 400–1000 nm, generating full spectral profiles for each pixel in the scene. Figures 10 and 11 show the mean raw intensity curves and the corresponding average normalized diffuse reflectance spectra acquired by the HS camera, providing a detailed comparison of the optical reflectance behavior of the polyamide, wood, and metal samples across the measured wavelengths.

As presented in Fig. 11, the normalized reflectance curves of the three studied materials reveal distinct behaviors across the spectral range. The white polyamide polymer and the wood sample show notable spectral structure, while the metal curve generally maintains higher reflectance in the visible range but flattens in the NIR.

- Polymer vs. wood separation: In the range of approximately 480 nm, the polymer exhibits higher relative reflectance compared to wood. This difference, although moderate, is significant enough to be exploited in selective band imaging. Conversely, beyond 670 nm up to around 840 nm, the wood reflectance intensifies markedly, surpassing that of the polymer. These contrasting spectral behaviors indicate two key separation windows: one near 480 nm favoring polymer detection, and another near 840 nm where wood is more reflective.
- Discrimination from metal: The inclusion of the metal sample’s curve shows how metals generally maintain consistently high reflectance in the visible range, often mimicking polymers in intensity but lacking the subtle absorptive or scattering signatures that characterize organic-based wood or polyamide. This means that relying solely on broadband average reflectance would risk misclassification, highlighting the importance of spectral detail.

**Fig. 12:**
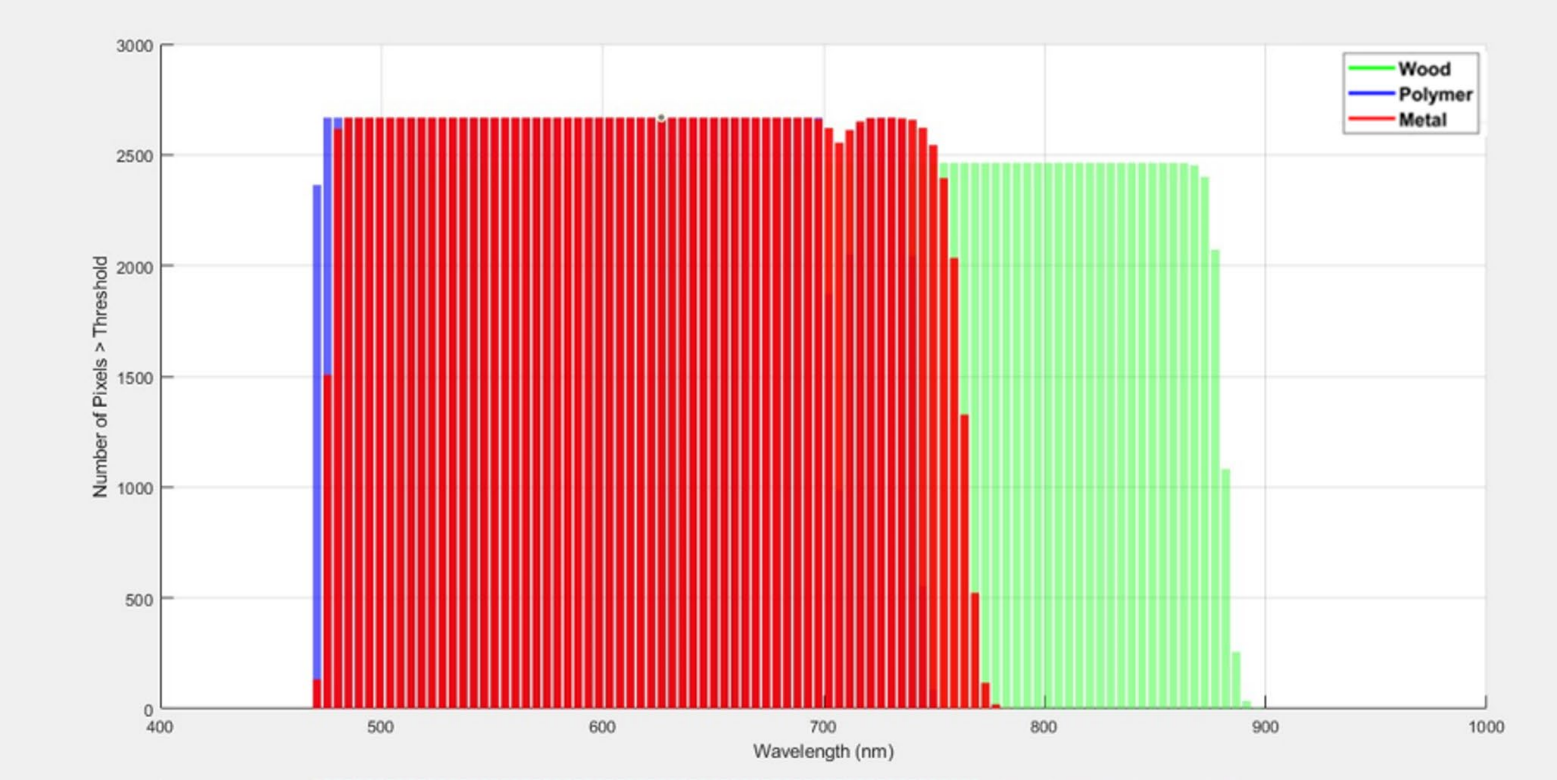
Histogram analysis showing the number of pixels exceeding a normalized reflectance threshold of 0.3 across the spectral range for white polyamide polymer, wood, and metal

To further quantify and localize these differences across spatial pixels and wavelengths, a threshold-based histogram analysis was conducted, as shown in Fig. 12. The normalized spectral values for each material were summarized using their mean and standard deviation to characterize distribution behavior, as depicted in Table 2. These parameters were computed from repeated measurements across the HS image cube, ensuring statistical reliability. The reflectance threshold of 0.3 was empirically determined through preliminary analysis of normalized reflectance distributions at 480 nm, a wavelength identified as optimal for polymer discrimination. This value lies above the region of significant spectral overlap (below 0.25), particularly between metal and polymer as observed in the normalized reflectance curves. By setting the threshold at 0.3, sufficient pixel representation was preserved to enable meaningful statistical analysis, while simultaneously maximizing inter-material discrimination. This balance ensures practical utility by reducing misclassification risk without excessively discarding relevant data. Furthermore, this combines spectral and spatial information, allowing us to see not just average curves but how consistently entire regions of the sample exceed meaningful optical response levels.

**Table 2:** Statistical parameters (mean and standard deviation) of normalized HS diffuse reflectance intensity distributions for the tested materials

Material	Mean	Standard deviation
Wood	0.46203	0.32703
polyamide polymer	0.35266	0.33297
metal	0.35988	0.32126

As shown in Fig. 12, three colored profiles represent the pixel counts across wavelengths for polymer, wood, and metal. The peaks in these distributions correspond to wavelengths where a substantial portion of each material’s surface exhibits strong reflectance relative to the chosen threshold (0.3).

- For the polymer, a clear peak emerges around 480 nm, indicating that a large number of pixels exceed the threshold at this wavelength, reaffirming the direct reflectance plot’s suggestion of polymer dominance here.
- For the wood sample, the histogram analysis showed that a relatively higher proportion of pixels exceeded the reflectance threshold (0.3) in the NIR region compared to the visible range, with the maximum separation from polymer observed near 840 nm. Although the histogram does not depict a sharp peak in the conventional sense, this indicates greater consistency of high reflectance values across wood surfaces in this region. This observation aligns with the normalized diffuse reflectance curves (Fig. 11), where wood reflectance progressively increases beyond 670 nm and remains higher than that of polymer through the NIR band, particularly around 840 nm.
- The metal sample exhibited consistently high reflectance across most of the visible range, resulting in relatively broad and elevated pixel counts in the histogram analysis. Unlike polymer and wood, however, the metal did not display distinct wavelength-specific peaks that could serve as strong discriminative features. Instead, its reflectance response remained relatively flat, lacking the dual-band separation observed in the polymer (480 nm) and wood (840 nm). This uniform behavior makes metal more challenging to classify using reflectance alone.

**Fig. 13:**
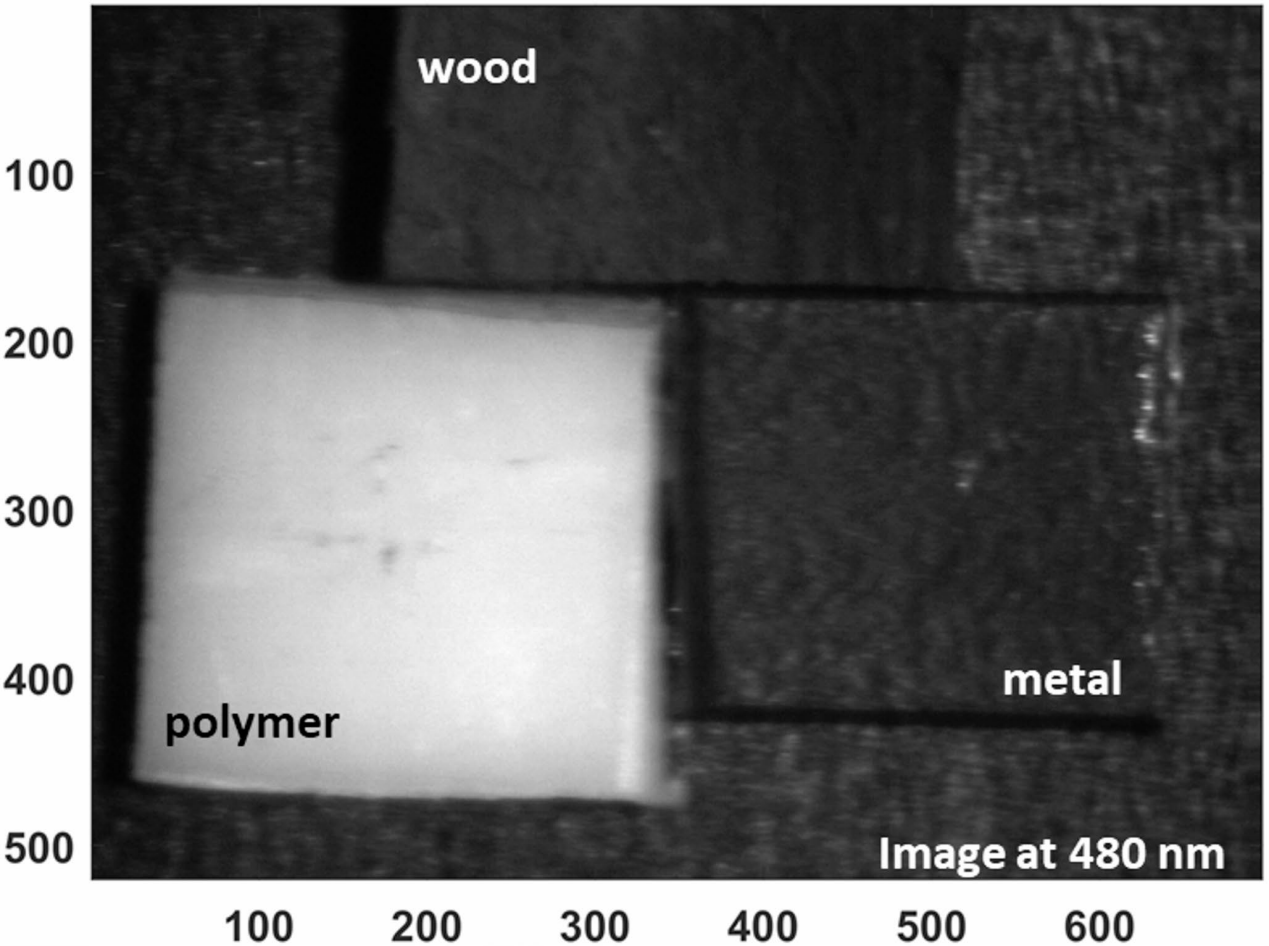
Hyperspectral reflectance image at 480 nm showing enhanced spatial contrast where the polymer exhibits higher reflectance relative to wood and metal

**Fig. 14:**
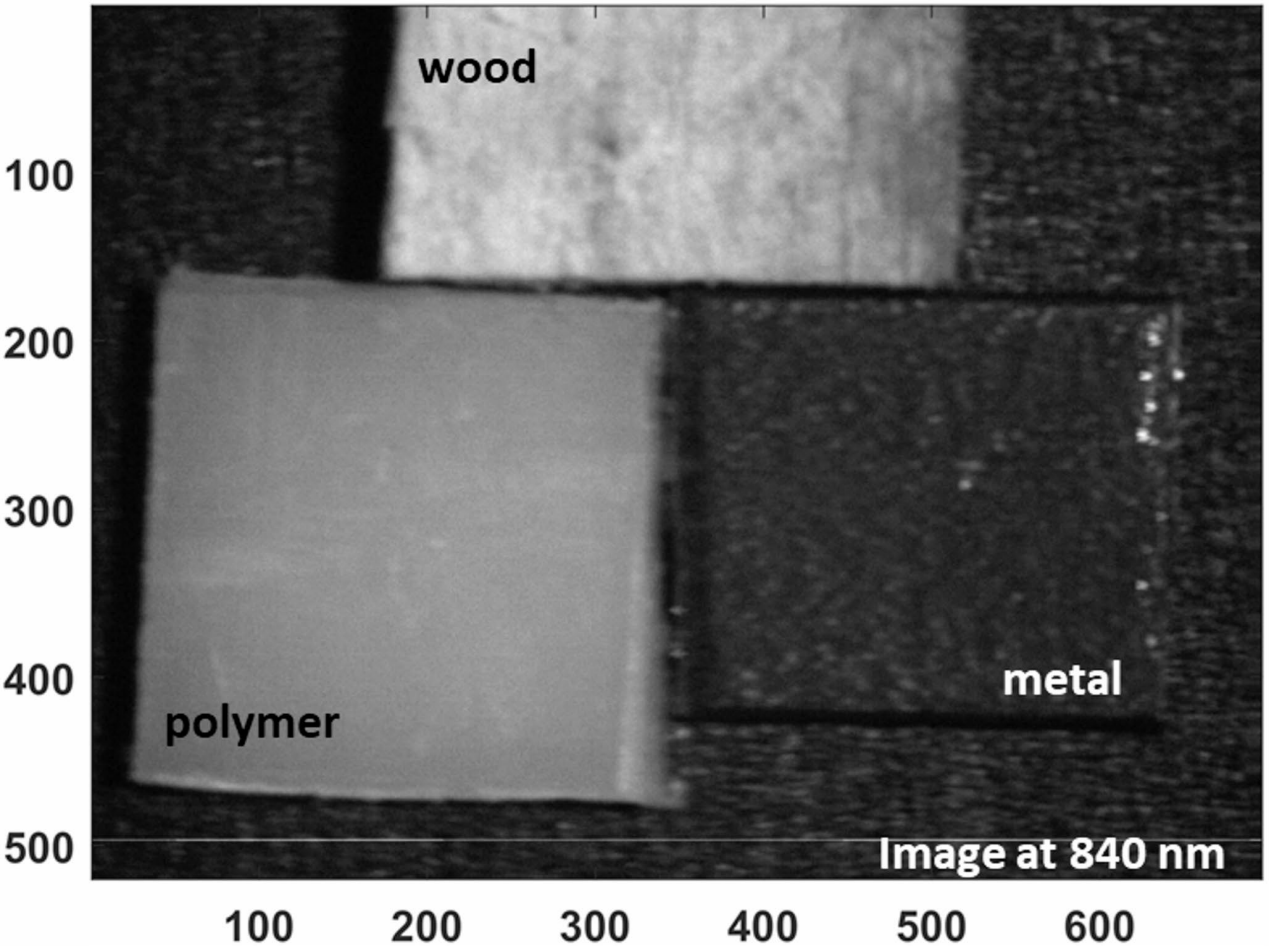
Hyperspectral reflectance image at 840 nm illustrating the dominant reflectance of wood in comparison to polymer and metal

This wavelength-dependent behavior optimizes material contrast, with 480 nm emerging as an optimal band for emphasizing the reflectance of the polymer relative to wood and metal, thereby facilitating effective clustering of the polymer pixels. In contrast, wood exhibits a pronounced increase in reflectance at 840 nm, while the polymer and metal samples maintain comparatively low activity at this wavelength. This distinct spectral separation positions 840 nm as an ideal wavelength for reliably identifying and classifying wood within mixed material streams. The effectiveness of these selected wavelengths is further illustrated by the corresponding hyperspectral images shown in Figs. 13 and 14, which visualize the spatial distribution and intensity differences at 480 nm and 840 nm, respectively. These images clearly demonstrate the enhanced discrimination achieved by targeting specific wavelengths tailored to the inherent optical properties of each material, underscoring the value of a spectrally informed approach in automated sorting applications.

**Fig. 15:**
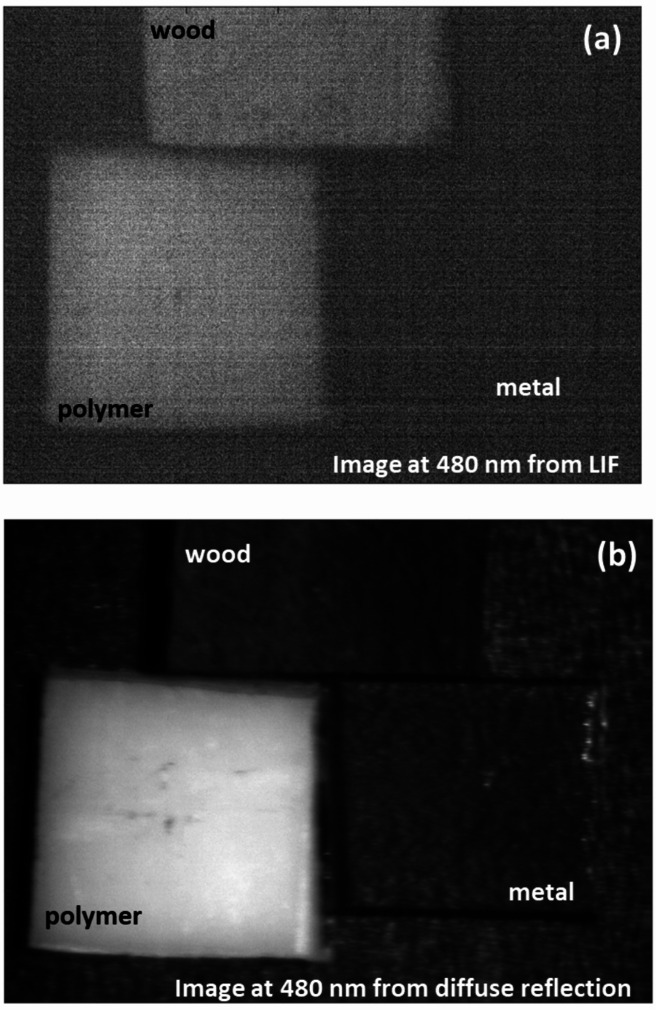
Comparison of spatial material contrast at 480 nm captured by the two imaging modalities: **a** laser-induced fluorescence, **b** diffuse reflectance hyperspectral imaging

By visualizing where peaks in pixel counts occur across materials, it enables precise selection of optimal wavelengths (480 nm and 840 nm) for implementing filtered camera systems. This is a key step towards developing simpler industrial systems that do not require full HS cameras but still leverage the identified critical bands. The integration of both LIF and diffuse reflectance HSI in this study underscores the necessity of employing a dual-modality approach for robust material discrimination. This is particularly evident when examining the spectral imaging outputs at 480 nm from both systems. In the fluorescence modality, the emission intensities of the polymer and wood are nearly indistinguishable at this wavelength, leading to poor spatial contrast and an inability to reliably separate these two materials. This limitation is visually confirmed by the fluorescence hyperspectral image at 480 nm (Fig. 15 (a)), where the overlapping signals of polymer and wood obscure clear classification. However, when assessing the same wavelength under diffuse reflectance conditions, the scenario is markedly different: the polymer exhibits significantly stronger reflectance than wood, resulting in high spatial contrast that enables effective discrimination (Fig. 15 (b)). This comparative analysis highlights why relying on a single imaging technique would be insufficient in complex waste streams where spectral overlaps occur.

**Fig. 16:**
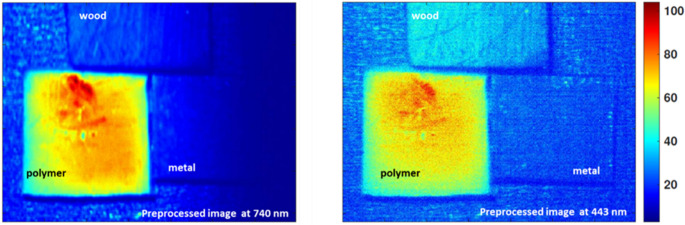
Effect of moving average filtering on LIF images at 740 nm and 443 nm prior to clustering

**Fig. 17:**
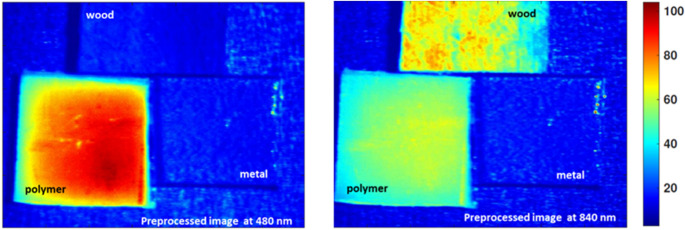
Enhanced diffuse reflectance images at 480 nm and 840 nm after moving average filtering, subsequent k-means clustering for automated material discrimination

As shown in Fig. 15, by combining these complementary modalities, the system achieves a more comprehensive optical characterization. This ensures that even when one technique encounters spectral ambiguity, the other can compensate, ultimately enhancing the reliability and accuracy of automated sorting processes. Building upon this foundation, we then advanced to the critical phase of exploiting the identified optimal wavelengths from both imaging approaches through a carefully tailored image processing framework. Specifically, we employed moving average filtering to effectively suppress high-frequency noise and enhance signal contrast, thereby sharpening material boundaries and improving the clarity of subtle spectral differences. Figure 16 illustrates this improvement vividly for the selected fluorescence outputs at 740 nm and 443 nm, where the polymer’s emission features become more pronounced against background variability. Similarly, Fig. 17 highlights the enhanced quality of the diffuse reflectance images at 480 nm and 840 nm, wavelengths chosen for their strong discrimination of polymer and wood respectively. This preprocessing not only refined the visual separability of the materials but also laid a robust groundwork for the subsequent application of unsupervised k-means clustering, enabling precise grouping of pixels by material class and thus advancing the automated sorting objectives of this study.

**Fig. 18:**
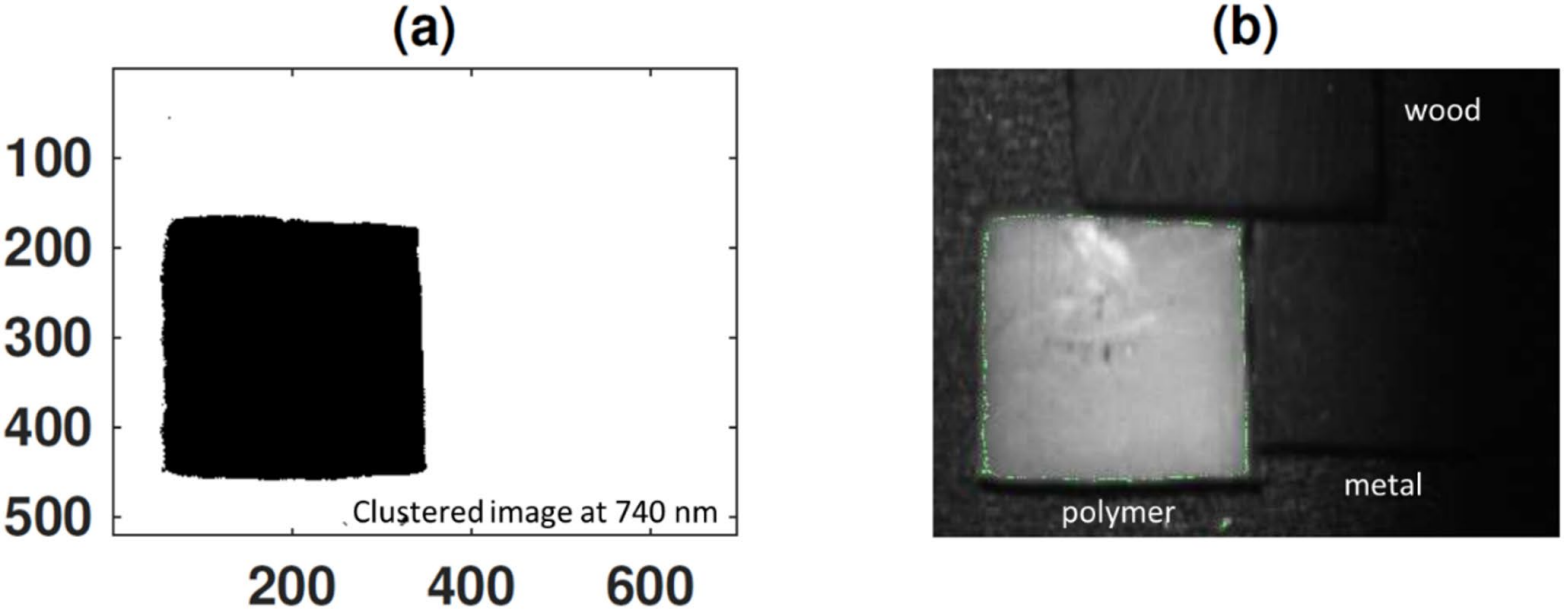
K-means clustering segmentation applied to the HS fluorescence image at 740 nm for isolating the polymer region. **a **Binary classification mask highlighting the polymer cluster distinctly separated from the surrounding materials. **b** Overlay of the polymer mask on the original grayscale fluorescence image, with the polymer area delineated in green, demonstrating precise localization driven by its unique fluorescence signature

**Fig. 19:**
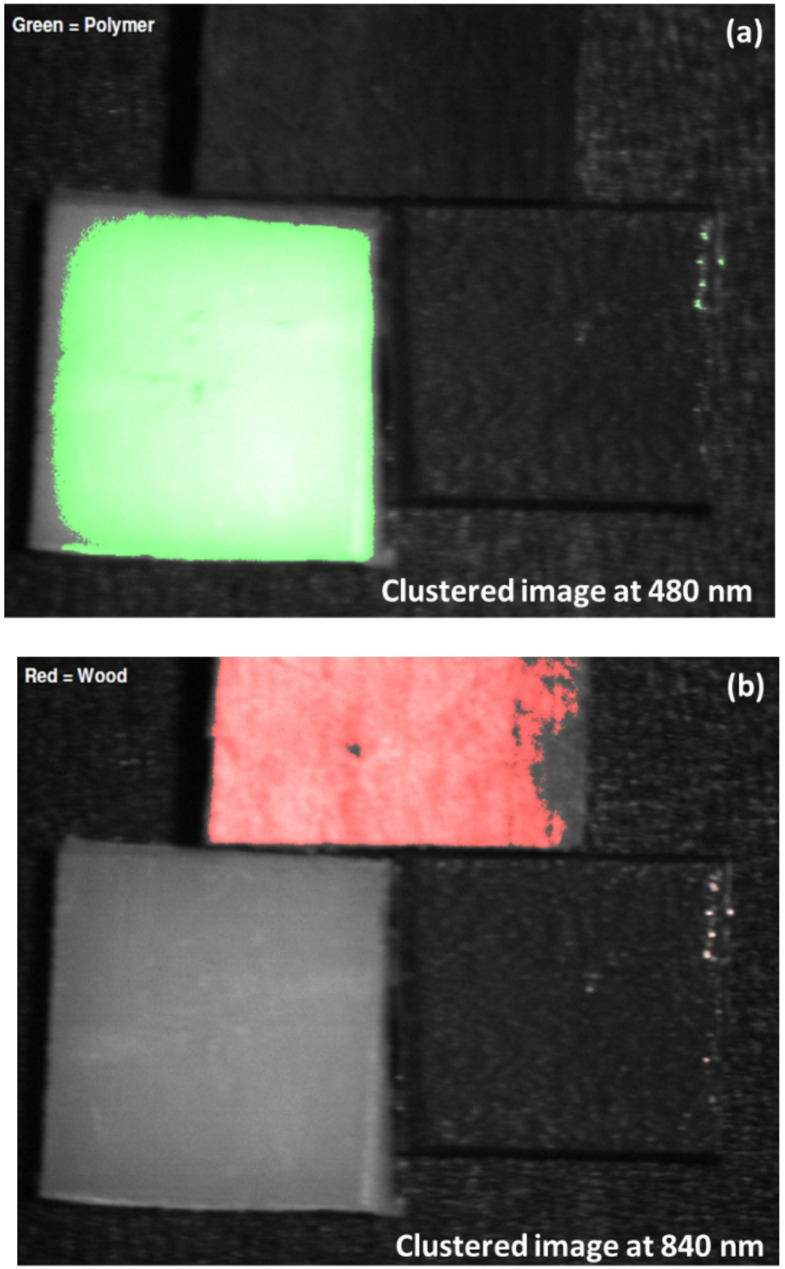
Final k-means clustering results for the diffuse reflectance images at 480 nm and 840 nm, illustrating effective material segmentation. **a** Polymer regions distinctly classified and shown in green. **b** Wood regions accurately identified and highlighted in red

Following this targeted preprocessing, we applied unsupervised k-means clustering to effectively segment and classify the materials of interest based on their enhanced spectral characteristics. This clustering was performed on the preprocessed fluorescence-based image at 740 nm (as shown in Fig. 16) to exploit the pronounced emission signature of polyamide polymer, as well as on the preprocessed reflectance-based images at 480 nm and 840 nm (Fig. 17), chosen for their demonstrated ability to discriminate white polymer and wood respectively. The clustering algorithm operates by iteratively partitioning the pixel data into three groups, minimizing the intra-cluster variance while maximizing inter-cluster separability, thereby enabling clear isolation of the distinct material classes present in the scenes [30, 36, 42]. Figure 18 illustrates the clustering results for the fluorescence modality, demonstrating how this approach sharply delineates the polymer from both wood and metal due to its unique emission peak at 740 nm. Conversely, Fig. 19 highlights the clustering outcomes for the diffuse reflectance images (480–840 nm), where the selected wavelengths successfully separate polymer and wood by capitalizing on their divergent reflectance behaviors.

As shown in Figs. 18 and 19, the resulting superimposed classification masks confirm that integrating the chemical specificity inherent to fluorescence imaging with the robust surface characterization offered by diffuse reflectance leads to markedly improved accuracy and reliability in sorting complex waste mixtures even under challenging conditions. This dual-modality clustering strategy not only strengthens material discrimination under conditions where individual techniques might falter but also underscores the promise of combining complementary optical signatures to achieve precise, automated identification of challenging recyclables in real-world applications.

## Discussion

**Fig. 20:**
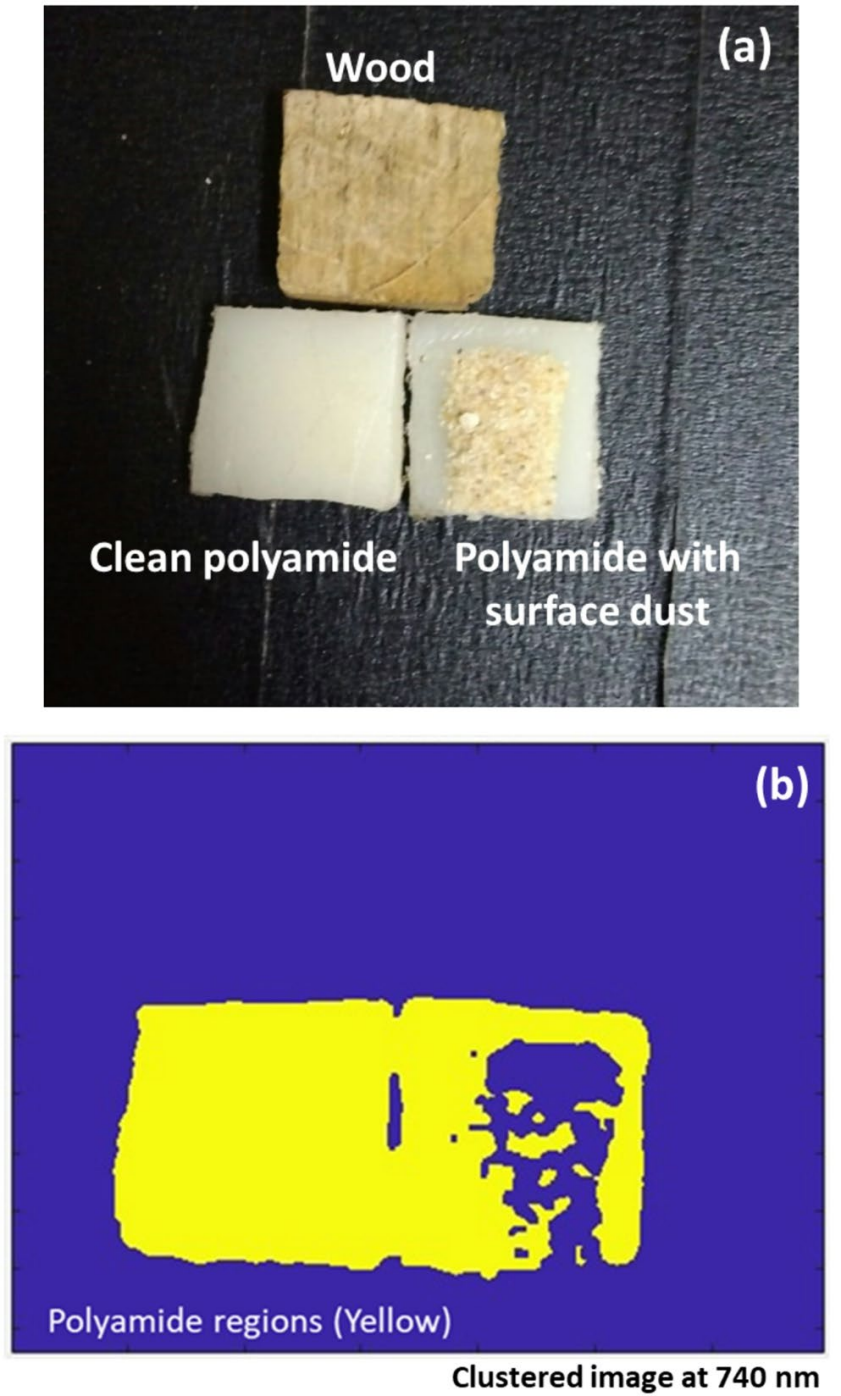
**a** Test samples used for experimental verification, including clean white polyamide, dust-contaminated white polyamide, and softwood with similar dimensions; **b** Clustering results at 740 nm fluorescence band using the LIF modality and k-means algorithm, demonstrating successful grouping of clean and contaminated polyamide regions while separating them from wood

The effective separation of visually similar materials remains one of the most persistent challenges in the field of automated recycling. Particularly troublesome is the case of light-colored engineering polymers such as white polyamide, which often exhibit strong visual resemblance to natural woods and even to oxidized or coated metals. This resemblance under typical illumination conditions frequently leads to misclassification in systems relying on conventional RGB imaging. Consequently, contamination of recycled polymer streams and inefficient recovery of valuable resources remain common, undermining both environmental goals and economic incentives. To address these limitations, this study explored a dual optical sensing strategy: combining LIF imaging with diffuse reflectance HSI. The fundamental rationale for this multimodal approach is grounded in exploiting two distinct physical mechanisms, molecular fluorescence under UV excitation, and broadband reflectance behavior under white light illumination. Whereas LIF leverages the unique emission properties of polymer molecular structures (such as amide groups in polyamide), diffuse reflectance HSI captures subtle differences in scattering and absorption due to variations in surface morphology and composition. A critical aspect of the diffuse reflectance HSI study was the use of histogram analysis. By quantifying the number of pixels exceeding a chosen reflectance threshold across wavelengths, the analysis revealed precise spectral regions where the materials diverged most significantly. This is particularly powerful because it moves beyond mean spectra alone, incorporating spatial heterogeneity in the sample responses. In practice, this histogram-based method identified optimal wavelengths near 480 nm (where the white polyamide exhibited peak separation from metal) and around 840 nm (where wood responses stood apart), thereby guiding future design of wavelength-selective filters or narrowband sensor configurations for rapid industrial sorting. Importantly, the findings also demonstrate why relying on a single imaging modality is insufficient in such scenarios. If only diffuse reflectance HSI were used, the inherent overlap in the visible region between white polyamide and wood would lead to ambiguities, especially under varied surface contamination or illumination inconsistencies. Likewise, using only LIF would miss critical reflectance cues; while the fluorescence peak of white polyamide near 740 nm offered robust discrimination, the nearly overlapping emission between wood and polymer in the 480–630 nm range underscores the need for complementary spectral domains. This dual imaging approach thus creates a more resilient classification landscape by uniting fluorescence-based molecular specificity with reflectance-driven surface contrast. To further demonstrate the practical potential of the proposed fluorescence-based approach, an experimental verification was conducted using three representative samples: (i) clean white polyamide, (ii) white polyamide contaminated with surface dust, and (iii) untreated softwood polished to a smooth matte finish, all having the same dimensions described previously (Fig. 20a). The surface dust used in this study refers to a thin, visually perceptible layer of fine household dust particles (< 0.5 mm thickness) manually deposited on the polyamide surface to simulate realistic environmental contamination conditions. The LIF modality was applied under the same acquisition and preprocessing conditions, followed by unsupervised k-means clustering for classification. Remarkably, the clustering results at the 740 nm fluorescence band successfully grouped both clean polyamide and polyamide regions from the dust-contaminated sample into the same class, clearly separating them from wood despite the presence of surface contamination, as depicted in Fig. 20b. These findings highlight the robustness of the proposed method in preserving material discrimination capability under real-world variability. Table 3 highlights how the proposed dual-modality strategy overcomes core limitations of traditional NIR-based polymer sorting, delivering richer data that is particularly vital for challenging cases like white polyamide against light wood or coated iron.

**Table 3:** Comparison between conventional NIR spectroscopy approaches and the proposed dual-modality imaging system combining LIF and diffuse reflectance HSI

Feature	Conventional NIR Spectroscopy	Dual-Modality LIF + HSI Approach
Spectral range	Primarily >900 nm	400–1000 nm (visible +NIR + fluorescence)
Sensitivity to white polyamide	Often low due to weak overtone absorption in NIR for polyamide	High due to direct fluorescence emission and visible reflectance differences
Material specificity	Limited for visually similar materials (wood vs. white polyamide)	Strong: molecular LIF signature + reflectance profile combined
Resistance to surface contamination/coatings	Moderate; absorption may be altered by dust or paint	Better resilience via dual mechanisms (fluorescence less affected by surface dust)
Data dimensionality for analysis	Low to moderate (few bands/single point)	High: enables pixel-wise histogram or spatially resolved clustering

**Fig. 21:**
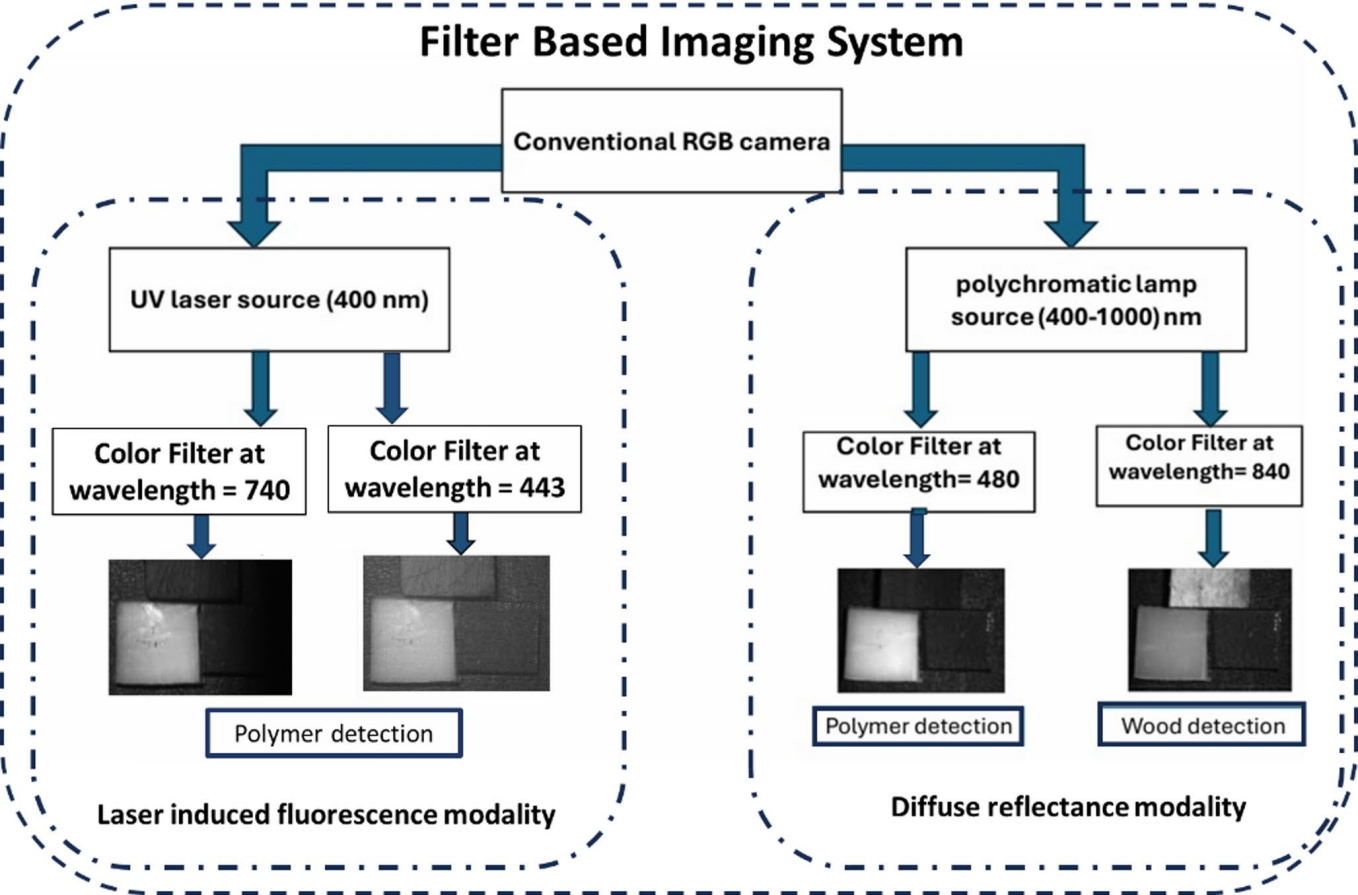
Proposed simplified filter-based imaging configuration for automated detection of white polyamide and wood in mixed waste streams, utilizing a conventional RGB camera equipped with discrete optical filters at 443 nm and 740 nm for LIF-based detection, and 480 nm and 840 nm for diffuse reflectance-based discrimination

Taken together, these findings carry important implications for the development of future automated recycling systems. The dual imaging framework enhances immediate material discrimination and establishes a foundation for designing simplified, cost-effective sensor arrays. For the diffuse reflectance modality, the analysis identified 480 nm as an optimal wavelength for detecting white polyamide and 840 nm for distinguishing wood. In parallel, the LIF results highlight 740 nm as the primary discriminative band, complemented by a secondary feature at 443 nm under 400 nm UV excitation. These wavelength selections can be directly implemented in filter-based configurations for conventional cameras, paired with controlled UV and broadband illumination sources, as depicted in Fig. 21. By clearly separating the contributions of each modality, this approach not only serves as a proof of concept for high-resolution spectral-based sorting but also provides a practical roadmap toward scalable, intelligent sorting platforms aligned with circular economy objectives.

While the proposed dual-modality approach demonstrates strong potential for discriminating visually similar materials under controlled conditions, several limitations must be acknowledged. A more rigorous evaluation using larger datasets, cross-validation, and classification performance metrics is needed. Future work will therefore focus on expanding the sample diversity, introducing environmental variability, and implementing advanced machine learning models for real-time classification. Furthermore, the integration of this approach into industrial sorting systems presents challenges related to illumination control, sensor cost, and processing speed, which will be addressed through optimized filter-based designs and hardware acceleration techniques.

## Conclusion

This study demonstrated a dual imaging strategy that leverages laser-induced fluorescence and diffuse reflectance hyperspectral imaging to address one of the most persistent challenges in automated recycling systems: the reliable differentiation of white polyamide polymers from visually and spectrally similar materials such as natural wood and oxidized or coated metals. By systematically evaluating these two complementary optical modalities under controlled conditions, we identified key spectral bands that enable clear discrimination, with fluorescence peaks at 740 nm and diffuse reflectance features around 480 nm and 840 nm emerging as optimal markers for material separation. The adoption of this dual imaging approach capitalizes on the molecular sensitivity of LIF alongside the broad spectral profiling of HSI, offering significant improvements over conventional single-modality or purely NIR-based methods that often fail to resolve subtle differences in chemically or physically similar waste streams. Importantly, histogram analysis proved to be a practical tool for pinpointing the most distinctive wavelengths in the reflectance domain, paving the way for simplified implementations using standard RGB cameras equipped with application-specific optical filters. This creates promising opportunities for translating these laboratory insights into cost-effective, scalable solutions, including aerial or conveyor-mounted camera systems for real-time waste monitoring and sorting. Altogether, the results not only advance the optical discrimination of challenging polymer types but also contribute to more efficient, cleaner recycling processes, supporting broader circular economy and sustainability objectives.

## Data Availability

No datasets were generated or analysed during the current study.

## Code Availability

The authors stated and declare that all code exists and is available.

## Declarations

All the co-authors are agreed for the research study publication.
